# A network website - The RENORBIO experience

**DOI:** 10.1186/1753-6561-8-S4-O45

**Published:** 2014-10-01

**Authors:** Paula Lenz

**Affiliations:** 1 RENORBIO, Universidade Estadual do Ceará, Fortaleza, Brazil

## 

The Brazilian Northeast, occupying an area of 1,554,387,725 km^2^, with approximately 3,000 km of coastline, has 56.46 % of its territory (877,565,831 km^2^) in a semi-arid zone [1], a region with unique climate and biodiversity in the world, with a very promising scenario for Biotechnology. However, it is a very poor region, concentrating 61.3 % of its municipalities with Low Human Development Index, the lowest rate in Brazil [2]. It is also a region with low capacity to generate wealth, contributing with only 13.5% of the Brazilian GDP [3] and 7% of the biotech companies in the country ([4]. In terms of skilled manpower [5], the Northeast is the third region with the lowest number of PhD researchers (18,433) and the second in numbers of PhDs per 100,000 inhabitants (34,77).

Nowadays, Biotechnology, besides the sustainable use of biodiversity, can make genetic modifications and breeding, including to genetically change the existing life forms, thereby leading to improved technology for the development and installation of the human species on the planet. Thus, this area progress can be accelerated if the government, the scientific community and the business community combine efforts in the development of joint projects, formation of productive partnerships, human resources training, creation of favorable environment for new investments and development and/or adaptation of technologies aiming to increase the competitiveness and to boost the market of biotech products. That is what the Northeast Biotechnology Network (RENORBIO) seeks.

Founded in 2004, the Network proposal is to integrate the scientific competence of the Northeast in biological sciences, based on a PhD course in Biotechnology (PPGB-RENORBIO), in order to enable a gradual rise of the competence dispersed in smaller universities that isolated, in the short or medium term, would not be able to create competitive graduate studies programs. The dispersed competences were replaced by a participatory model that promoted the integration of the critical mass of Northeast professors and researchers to qualify PhDs in Biotechnology, with the formal support of the institutions involved and the Science and Technology Departments of the States of the Northeast and Espírito Santo. Therefore, several structural actions have been performed, some already established, others in progress, combining cost reduction with parallel and redundant investments and consequent gradual consolidation of Centers of Excellence in Biotechnology in the Northeast.

In 2006, together with the structure of the PPGB-RENORBIO, the construction process of a Website (http://www.renorbio.org.br), which would meet the needs of a network program, involving, at the time, 28 institutions distributed in 10 states, also began. During its first five years, PPGB-RENORBIO academic process, of all its students, was through this website: from the selection process, subject offerings and enrollment, subject communities, to reports and data collection of professors and students from Lattes CV. The Website was therefore an important tool in the construction of RENORBIO identity, meeting the network basic principle, i.e., to eliminate institutional, local visions, in favor of a more comprehensive regional vision, in order to enable the Northeast to grow as a whole through Biotechnology.

RENORBIO today includes 36 institutions, 246 professors and 520 students; supervises 15 post- doctorates; 281 PhDs were qualified. Five years after it started, the number of requests for patent applications increased from 8 to 216; the number of professors involved with patents increased from 6 to 70; in addition to the considerable increase in the production of scientific papers by its researchers and students; as well as the significant increase of publications in higher impact journals. For such a young biotechnology program, these data are of great importance, as it shows that there is a substantial change in the culture of creation of products/processes in the region. Now, RENORBIO is concerned with the effective transfer of innovative processes and products created in its laboratories in order to produce wealth, life quality and social inclusion in the Northeast. A challenge that has just began to be conquered by the creation of the first 11 companies constituted by students, graduates and professors, as well as the licensing of 17 inventions.

RENORBIO website greatly contributed to obtain these promising results in several ways: through its integrating role, giving a unique identity to RENORBIO; its management potential, providing data for management; and its disclosure ability, giving visibility both nationally and internationally to the program.

The website integration can be best understood by considering the program logic. RENORBIO chose to use its own system, secure, with a design that emphasizes its logo and completely different from any of the participating institutions website. The idea was to eliminate any merely institutional or multifaceted vision of the Network and to introduce the concept of a single regional program with a roving coordination. Thus, all academic procedures of the students, from 2006 to 2011, were conducted in RENORBIO Website and the data transferred to the institutions responsible for the certificate issuance. As of 2012, new procedures were adopted, as described below.

As an excellent tool used in multi-institutional projects, the website consists of records of professor and student and administers the selection process, online registration of students, subject offers, academic transcripts, records of professors, student reports, among others, additionally to the Center for Teaching Support (NAE). NAE is the space where professors and students meet at Renorbio Graduate Studies Center. There, each subject consists of a community of practice, unique, that integrates all students and professors connected to it with a set of opportunities available that support the professors' various tasks, such as issuance of grades and attendance, paper request and receipt, bulletin board, conduction of surveys, among others. To students, in addition to the features related to the classroom, NAE provides means to interact with peers and the professor, enabling communication among them - chat room and messaging tools - as well as providing information on the academic life.

As a management tool, the website is a database containing all the information about the students and those relevant for the professor's program. The manager has full access to the Website, he/she may enter any information or modify any data whatsoever, and therefore, the PPGB-RENORBIO Executive Secretary was the only one with this access. However, all information can be removed through specific reports, without interfering with the quality of the data, thus allowing all further members of the program management to have the information they need directly from the Website.

The difficulty of gathering information from such a large group, about 600 people active per year, needs to be facilitated for the manager. Thus, mechanisms to collect data from professors and students were created, and, among them, the capture of information of interest from the curriculum vitae on CNPq Lattes stands out. Throughout time, other requirements not initially foreseen were identified and some of them have already been developed, while others are currently under development.

As of 2012, a new enrollment procedure was established and each student started to enroll directly into the website of the institution where he/she belongs. However, in case the student wishes to attend subjects offered by other institutions of the Network, RENORBIO Website should still be used for enrollment. The new challenge is to maintain, in this new structure, RENORBIO identity/unity already conquered. Some important actions may be: to provide a copy of each student's updated academic transcript, to give continuity and to improve the students' semi-annual reports and to create a space for annual recertification of the professors, as well as to maintain and improve internal demand mechanisms.

In addition to the strong use of the website by RENORBIO members, it seems that it has promoted good visibility to the Program and provoked the interest not only of national groups, but also international. From mid-2010 until the present time, 145,814 people visited RENORBIO website, accessing an average of 5 pages per visit, with an average duration of 4 minutes and bounce rate of 46.54%. The percentage of new visitors was 43.8 %, and the return rate was 56.2 %. At a national level, the website was visited by more than 250 municipalities from several Brazilian states, but mainly by the northeastern capitals and the states of Rio de Janeiro, Minas Gerais and São Paulo. Internationally, countries such as the United States, Germany, Colombia, Spain, France, Argentina and Italy are among the 10 who accessed RENORBIO website the most over the last month, with a median of 232 visits, 67.76 % new visits, median rate rejection of 49.96 %, an average of 4 pages per visit, with an average duration of 3 minutes. Portugal and the United States accounted for 1430 and 1370 visits, respectively, but with the highest rejection rates (73.85% and 64.45%, respectively). Colombia had 242 visits, with 68.60 % new visits, the lowest rejection rate (38.02 %) and access to a high number of pages (6.86), with a visit average duration of 7min. These data are consistent with the increasing number of foreigners interested in applying to the PhD at the PPGB-RENORBIO. In 2012, three Colombians and one French were accepted.

RENORBIO website also has space to publicize the labs that integrate the Network, the biotechnologies developed by students and/or professors and the opportunities of scholarship/jobs/internships, which, still, need to be better explored.
